# Shifts in Small Fish Community Caused by Coral Cover Variation at a Remote Reef in the South China Sea

**DOI:** 10.1002/ece3.73553

**Published:** 2026-04-23

**Authors:** Daxiao Ling, Xianzhi Lin, Yanyan Zhou, Yang Liu, Haoye Lin, Li Zhang

**Affiliations:** ^1^ State Key Laboratory of Tropical Oceanography, Guangdong Provincial Key Laboratory of Applied Marine Biology, South China Sea Institute of Oceanology Chinese Academy of Sciences Guangzhou China; ^2^ University of Chinese Academy of Sciences Beijing China; ^3^ Sanya Joint Laboratory of Marine Science Research, Key Laboratory of Tropical Marine Biotechnology of Hainan Province Sanya Institute of Ocean Eco‐Environmental Engineering Sanya China

**Keywords:** assemble mechanisms, biodiversity, coral reefs, environmental DNA, underwater visual census

## Abstract

Global coral reef fish biodiversity is undergoing a severe decline driven by both natural and anthropogenic disturbances. However, the biodiversity and ecological functions of small fishes have long been underestimated, mainly because small fishes are difficult to capture or to identify using underwater visual census (UVC). This study integrated environmental DNA (eDNA) metabarcoding and underwater visual census (UVC) to systematically survey the small fish community at Meiji reef, a region comprising two distinct habitats characterized by high coral cover (HC) and low coral cover (LC). A total of 149 small fish species were detected by the two methods combined, with eDNA identifying 132 species and UVC only 38. Species richness, Shannon index, and phylogenetic diversity of small fish are lower in LC, while functional diversity is higher in HC, suggesting that species retention is critical to maintaining ecosystem functions. Nevertheless, the overall decline in functional redundancy indicates a weakened buffering capacity and reduced resilience. Network analysis further revealed that HC areas exhibited denser node and connection structures and higher modularity. Correspondingly, the indicator species transitioned from Pomacentridae and Clupeidae in HC areas to benthic Blenniidae in LC areas, a taxonomic manifestation of ecosystem simplification resulting from the loss of structurally complex coral habitats. As coral cover decreased, the assembly mechanism of the small fish community shifted from a combination of environmental filtering and stochastic processes to stochastic dominance. Furthermore, significant interactions among eco‐environmental factors and the biological community collectively shaped species distribution patterns, driving divergence in small fish community structures across regions. In summary, this study elucidates the mechanisms by which small fish communities respond to coral cover gradients at a fine habitat scale, providing essential insights into the ecological functions and environmental adaptability of small coral reef fish species.

## Graphical abstract

We conducted a systematic survey of the small fish community using eDNA metabarcoding and UVC at Meiji Reef, a remote coral reef in the South China Sea with rich coral habitats. Our results confirmed the superior detection capability of eDNA for small fishes. Collectively, our findings illustrate how declining coral cover reshapes the small fish community structure and assembly by weakening environmental filtering while strengthening stochastic dispersal, ultimately driving the ecosystem toward a simpler and potentially unstable state. This study provides essential insights into the ecological functions and environmental adaptability of small coral reef fish species.

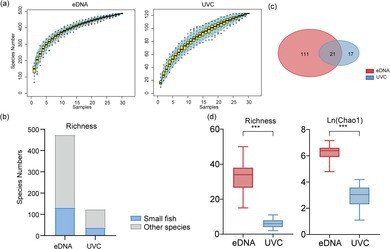

## 1 Introduction

Tropical coral reefs harbor approximately one‐third of the world's marine fish biodiversity, ranking among the most species‐rich ecosystems on Earth (Spalding and Grenfell 1997; van Woesik et al. 2022). However, these ecosystems face persistent threats of degradation from environmental stressors driven by both natural and anthropogenic processes, leading to declining diversity and an increasing proportion of small‐sized species within coral reef fish communities (Zhang et al. 2024; Zhao et al. 2024). Over the past decades, research on coral reef fishes has predominantly focused on large, economically valuable species, while the distribution patterns and ecological functions of small fish have been a relatively neglected component of reef ecosystems, resulting in a limited understanding of the roles in ecosystem functioning and overall stability.

Defined as species with a maximum recorded adult body length of less than 10 cm (Brandl et al. 2024), small fish species consume detritus and plankton, thereby channeling energy to higher trophic levels and substantially enhancing the productivity of coral reef ecosystems (Maie et al. 2009; Mihalitsis and Bellwood 2017; Ruzicka et al. 2024). Their lower mobility and more limited dispersal compared to larger species promote higher species richness at small spatial scales (Depczynski and Bellwood 2006; Taylor and Hellberg 2005; Wagner et al. 2019). Minor habitat differences can create practical geographic barriers, while rapid generational turnover facilitates the accumulation and retention of genetic variation, thereby promoting rapid speciation (Taylor and Hellberg 2006; Tornabene et al. 2015). Although many small fish species exhibit r‐strategy traits that may enhance their resilience, their strong reliance on specific habitats can still make them highly sensitive to habitat degradation, particularly in coral reef ecosystems. (Brandl et al. 2016). In addition, their short life cycles and specialized niches consequently render them highly sensitive to environmental disturbances, including thermal anomalies and ocean acidification (Hughes et al. 2017; Murray et al. 2019). Therefore, they serve as valuable bioindicators of coral reef ecosystem health, often exhibiting earlier and more pronounced responses to environmental perturbations than larger species (Debnath 2025). Consequently, a deeper understanding of the ecological roles and behavioral mechanisms of small fish is essential both for advancing fundamental science and for informing effective management strategies for coral reef ecosystems.

In situ survey methods, including underwater visual census (UVC) and fishing‐based sampling methods, may face inherent challenges in detecting and identifying small fish species. Small fish species often exhibit subtle, overlapping interspecific morphological traits (e.g., coloration and behavior), making them challenging to locate and collect effectively. Technical limitations have severely constrained the acquisition of reliable data worldwide. The standard method of combining self‐contained underwater breathing apparatus (SCUBA) diving with chemical anesthetics (e.g., clove oil) requires relatively calm water conditions (Brandl et al. 2017; Robertson et al. 2022). Underwater visual census (UVC) is generally more effective for detecting larger and more conspicuous fishes, but has significant limitations in identifying and recording small species (Ackerman and Bellwood 2000; Mathon et al. 2022). In recent years, the widespread application of DNA sequence data in taxonomy has gradually improved this situation (Courtaillac et al. 2025). Environmental DNA (eDNA) metabarcoding technology enables high‐throughput and highly sensitive detection of community biodiversity under non‐invasive conditions (Blackman et al. 2024), demonstrating significant efficacy in biodiversity assessment and rare species detection (Chen et al. 2024). To some extent, eDNA overcomes the challenges posed by the cryptic nature, difficulty of capture, and problematic identification of small fish, thereby providing novel methodologies and perspectives for their research and monitoring.

This study aims to examine how small fish communities respond to changes in coral reef habitats. Our research investigated small fish communities at Meiji Reef in the South China Sea to determine patterns of species diversity across coral reef habitats with varying health statuses. Among the eco‐environmental variables, we focused on coral cover as a key habitat characteristic, as corals are important habitat engineers whose structural changes directly and indirectly shape fish assemblages. We expected to clarify the spatial distribution patterns of the small fish community at Meiji reef and to elucidate the mechanisms underlying their response to different coral cover, providing evidence for understanding the ecological processes of small fish dynamics.

## 2 Materials and Methods

### 2.1 Field Investigation

**FIGURE 1:**
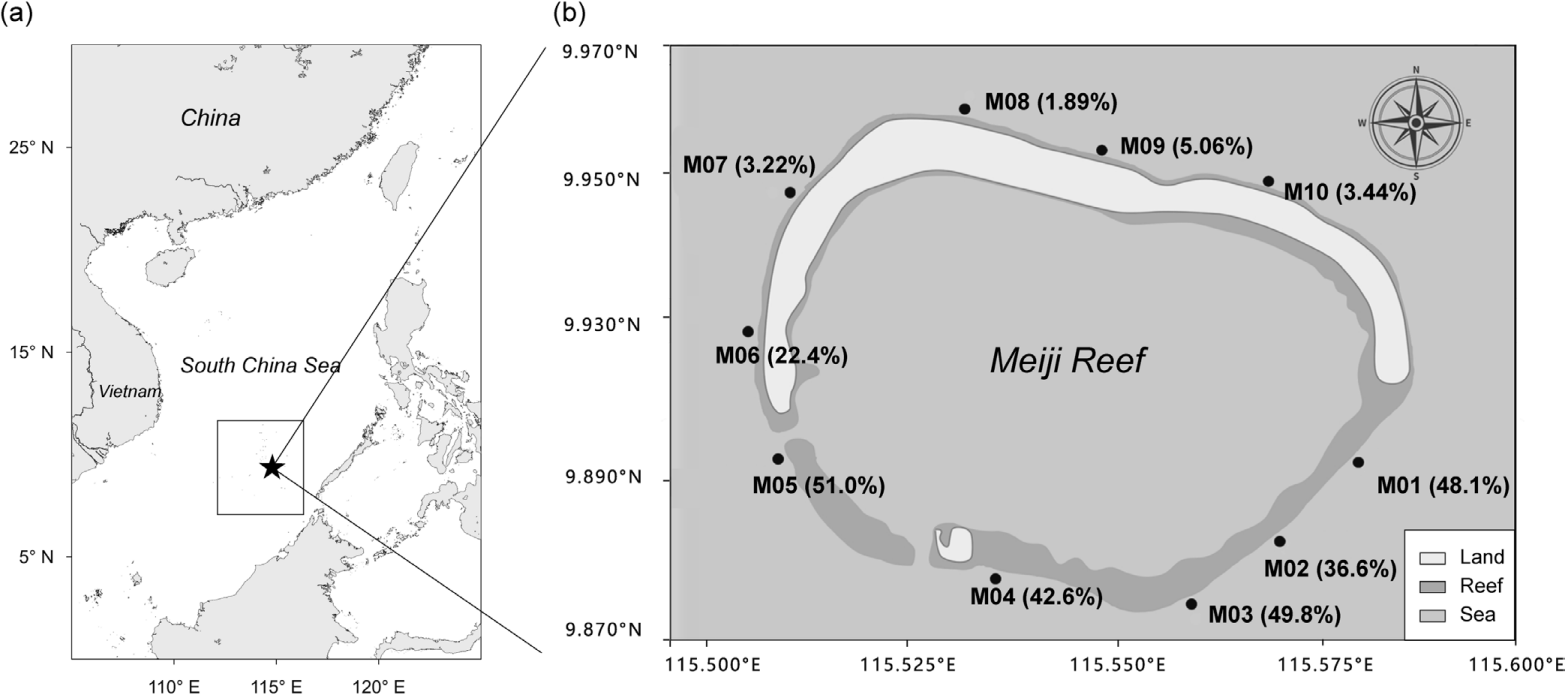
(a) The location of Meiji Reef atoll. (b) Ten sampling sites were strategically established around Meiji Reef. The data in parentheses represent coral coverage.

This study was conducted at Meiji reef in the southeastern South China Sea. Meiji reef is a remote tropical coral reef, characterized by two distinct habitats: high coral cover (HC, abbreviations used are listed in Table S2) in the southern region and low coral cover (LC) in the northern region (Lin et al. 2021). Ten survey sites were established in June 2023 along the outer slope of Meiji reef, encompassing both coral‐rich and coral‐depleted areas (Figure 1a,b).

Three replicates of 2 L surface water were collected at a depth of approximately 0.5 m using a water sampler from each site according to the national standards of the People's Republic of China: The Specification for Marine Monitoring (GB 17378.3‐2007). The samples were immediately filtered through 0.45 μm pore‐sized mixed cellulose ester (MCE) membranes to capture eDNA. The membrane filters were stored in 2 mL sterile cryotubes and preserved at −20°C before DNA extraction. To monitor potential cross‐contamination, three negative control samples were concurrently prepared by filtering 1 L of deionized water through each membrane. At each eDNA sampling site, environmental parameters, including temperature, salinity, dissolved oxygen (DO), and pH, were measured in situ using a YSI multiparameter water‐quality analyzer (YSI Incorporated, USA). Site depth was measured using a portable depth sounder (Speedtech, SM‐5, USA). Seawater flow velocity (mm/s) was measured on‐site using an acoustic Doppler velocimeter (Workhorse ADCP, TRDI, USA).

Concurrently, systematic surveys were conducted to quantify coral and other taxa coverage around each site. Each sampling site was established across three depth strata (3, 8, and 15 m), with three 60 m transects deployed per depth. A handheld underwater camera was used to record video at a constant speed along each transect. Video recording duration for each transect was standardized to approximately 10 min. The fish monitoring quadrats were first conducted, each spanning 60 m in length and covering a total survey area of 60 m × 5 m (extending 2.5 m on either side of the transect line). During observations, the investigator held a measuring scale at eye level, focused several meters ahead, and recorded the abundance of common fish species within a 2.5‐m range on both sides of the measuring scale. Body lengths were visually estimated and recorded using the scale and categorized into four size classes: < 10, 10–20, 20–40, and > 40 cm. During the UVC survey, high‐definition underwater cameras were used to record the fish community along each transect, particularly targeting small fishes that are highly mobile or difficult to identify in situ. After the survey, the recorded transect videos was reviewed and organized, and species that were difficult to identify in the field were re‐examined by taxonomic experts in conjunction with divers' field notes. This process allowed for verification and correction of species identifications, reducing potential errors associated with single on‐site observations.

After fish monitoring, benthic cover categories along the same transect line were recorded by underwater camera to characterize substrate conditions. The recorded transect videos were transferred to a computer for subsequent analysis. Substrate categories were identified at 10 cm intervals along each transect, and corresponding categories (e.g., reef‐building coral taxa and large benthic invertebrates) were recorded and compiled. The occurrence frequency of individual taxa (*N*) was quantified based on transect interpretation, and coral and large benthic invertebrates cover (*C*) was calculated as:

C=N/600×100%.

Fish individuals were caught using a trammel net (10 m long × 1.5 m high, with maximum and minimum mesh size of 9.0 and 3.0 cm, respectively), and were removed from the trammel net within an hour of being trapped.

### 2.2 Environmental DNA Extraction and Sequencing

The eDNA samples and blank controls were mechanically lysed using the FastPrep instrument (MP Biomedicals, USA) with the addition of silica sand, followed by homogenization at 6.0 m/s for 40 s. The homogenate was suspended in lysis buffer (containing 100 mM EDTA, 10 mM Tris‐Cl, 0.5% SDS, and 20 μg proteinase K) and incubated at 55°C for 24 h. DNA was extracted from the homogenate using a modified cetyltrimethylammonium bromide (CTAB) protocol (Zhang and Lin 2002) and phenol‐chloroform‐isoamyl alcohol extraction, and finally dissolved in 30 μL of 10 mM Tris–HCl (pH 8.0). DNA quality was assessed using a NanoDrop spectrophotometer (NanoDrop Technologies, Wilmington, DE, USA), and negative extraction controls were established to monitor contamination risks. All extracted DNA samples were stored at −20°C for further use.

The hypervariable region of 12S rRNA (Miya 2022) was amplified by polymerase chain reaction (PCR) using the primers Mifish‐U/F (5′‐GTCGGTAAAACTCGTGCCAGC‐3′) and Mifish‐U/R (5′‐CATAGTGGGGTATCTAATCCCAGTTTG‐3′). Each extracted eDNA sample was diluted to 10 ng/μL with sterile deionized water and used as a template for PCR amplification. PCR was performed in a 25 μL reaction system containing 10 ng of diluted DNA as the template, 1 μL each of Mifish‐U/F and Mifish‐U/R primers (10 μM), and 12.5 μL of 2 × Taq Mix (Tsingke Biotechnology Co. Ltd), with sterile deionized ddH_2_O added to a final volume of 25 μL. The PCR thermal profile consisted of an initial denaturation at 95°C for 5 min, followed by 35 cycles of denaturation at 95°C for 10 s, annealing at 57°C for 30 s, and extension at 72°C for 1 min, with a final extension at 72°C for 10 min. Negative controls were performed by substituting ultrapure water for DNA in each PCR. The PCR products were quantified and assessed for quality using the Qubit 2.0 Fluorometer (Thermo Scientific, Waltham, MA, USA) and 1% agarose gel electrophoresis, followed by purification using the AxyPrep DNA Gel Extraction Kit. No bands were detected in the negative controls for DNA electrophoresis, extraction, and PCR. Nevertheless, all negative controls were included in the sequencing to assess potential contamination further. The purified products were quantified using the Qubit 3.0 (Life Invitrogen), pooled in equimolar amounts, and used to construct Illumina paired‐end sequencing libraries according to the Illumina genomic DNA library preparation protocol. The amplicon libraries were sequenced on the Illumina Novaseq platform (BIOZERON, Shanghai) using the PE150 protocol according to standard procedures. The raw sequencing data have been deposited in the NCBI SRA database (Accession Number: PRJNA1366653), and the sequencing results show that the negative control is free from contamination. In the sequencing results, one of the three replicates for the M04 sample failed to achieve the desired abundance due to sequencing failure; consequently, this replicate was excluded from subsequent analyses.

### 2.3 Bioinformatics

Paired‐end sequencing reads were initially quality‐filtered and trimmed, with a threshold of a maximum expected error (maxEE) of 2 per read. The filtered reads were then dereplicated, and insertions/deletions (indels) and substitutions were detected using the DADA2 algorithm as recommended in QIIME 2. After merging paired‐end reads and removing chimeras, a feature table was constructed based on amplicon sequence variants (ASVs). Subsequently, each 12S rRNA gene sequence was assigned a phylogenetic affiliation using two databases, MitoFish (http://mitofish.aori.u‐tokyo.ac.jp/) and the National Center for Biotechnology Information (NCBI, https://www.ncbi.nlm.nih.gov/). A confidence threshold of 80% was used for taxonomic assignment, representing a balance between assignment accuracy and detection sensitivity. Ecological trait data—including body length and trophic guild—were extracted from the FishBase database (https://fishbase.de/) to aid in the delineation of small fishes and to explore community assembly patterns. Specifically, only ASVs assigned to the species level were retained, and sequences with counts of fewer than 10 per species were excluded. Fish species and family names were further cross‐referenced with FishBase to resolve synonyms. Additionally, species with a low probability of occurrence in the South China Sea, as indicated by FishBase distribution records, were excluded from the analysis. Species richness was rarefied with sequencing depth, and a rarefaction curve was plotted. ASV tables were normalized to the depth of the smallest library. All subsequent eDNA analyses were conducted in R (version 4.4.2) utilizing the rarefied and normalized ASV abundance table.

### 2.4 Statistical Analysis

Principal component analysis (PCA) was conducted using the factoextra and vegan packages to visualize multivariate patterns (Kassambara and Mundt 2020; Oksanen et al. 2012), facilitating classification of sampling sites into coral‐rich and coral‐depleted areas. Ecological trait data—including body length and trophic guild—were extracted from the FishBase database (https://fishbase.de/) to aid in the delineation of small fishes and to explore community assembly patterns.

The MEGA software was used to construct phylogenetic trees for all identified small fish species for subsequent analysis. Multiple diversity indices were calculated to evaluate community alpha diversity based on UVC and eDNA results: taxonomic diversity (TD) was quantified using the Shannon–Wiener index, computed with the vegan package in R. Functional diversity (FD) was assessed using the functional richness index (FRic), calculated from trait data using the dbFD function in the FD package. Phylogenetic diversity (PD) was determined by first constructing a phylogenetic tree from ASV sequences using the maximum likelihood method in MEGA11 (Tamura et al. 2021), then calculating the sum of branch lengths for each sample using the pd. function in the picante package (Kembel et al. 2010). Inter‐group comparisons of alpha diversity were conducted using Wilcoxon rank‐sum tests.

To investigate variations in community structure, we employed a multifaceted analytical approach. Species composition and abundance data obtained from eDNA metabarcoding were standardized using Hellinger transformation (Laporte et al. 2021). Compositional differences in the small fish community between HC and LC habitats were assessed using Bray‐Curtis and Jaccard dissimilarity matrices and visualized using principal coordinates analysis (PCoA). The statistical significance of group separations was tested using permutational multivariate analysis of variance (PERMANOVA) with 999 permutations. Indicator species characterizing each habitat type were identified using linear discriminant analysis (LDA) and effect size analysis (LEfSe), implemented via the trans_diff function in the R package microeco, with an LDA score threshold of 2.0 and a significance level of *α* = 0.05 (Khleborodova et al. 2024). Co‐occurrence networks were constructed and visualized using the R package psych and Gephibased on Spearman rank correlation coefficient (Mathieu Bastian 2009). The network's robustness metrics were assessed (Montesinos‐Navarro et al. 2017), and network stability was compared across community types based on the robustness metrics.

To elucidate underlying ecological processes, we applied the infer community assembly mechanisms by phylogenetic bin‐based null model (iCAMP) analysis to quantify the relative contributions of different community assembly mechanisms: homogenizing selection (HoS), heterogeneous selection (HeS), dispersal limitation (DL), homogeneous dispersal (HD), and ecological drift (DR) (Ning et al. 2020). Eco‐environmental drivers were analyzed using correlation analysis with the microeco package (Liu et al. 2021), retaining only statistically significant variables (*p* < 0.05) with correlation coefficients < 0.7 to avoid multicollinearity. Redundancy analysis (RDA) was performed using the vegan package to examine the relationship between remaining eco‐environmental factors and community structure. Eco‐environmental variables were Z‐score standardized before analysis. The significance of individual eco‐environmental factors was tested using the envfit function with 999 permutations, and adjusted *R* ^2^ values were computed using the RsquareAdj function; *p* < 0.05 was considered statistically significant. The linear regression statistics were calculated using the base R stats package via the lm() function for model fitting and the summary() function for extracting statistical parameters, reporting the coefficient of determination (*R* ^2^), regression slope, and significance level (*p* < 0.05). The distHaversine function in the geosphere package calculates distances on the Earth's surface. All images, except the co‐occurrence network, were plotted using the ggplot2 package (Coleman et al. 2023).

## 3 Result

### 3.1 Eco‐Environmental Conditions in Survey Areas

Ecological variables were recorded during the sampling period, including the eco‐environmental parameters and substrate cover for each site (Table S1). Based on coral cover, sites were classified as the high coral cover group (HC) and the low coral cover group (LC). As illustrated in Figure S1, coral cover differed between the HC and LC groups: ≤ 5% in the LC group and > 20% in the HC group. Variables such as water temperature, salinity, and dissolved oxygen showed limited variation across the 10 sampling sites (Table S1).

### 3.2 Diversity and Composition of Small Fish

**FIGURE 2:**
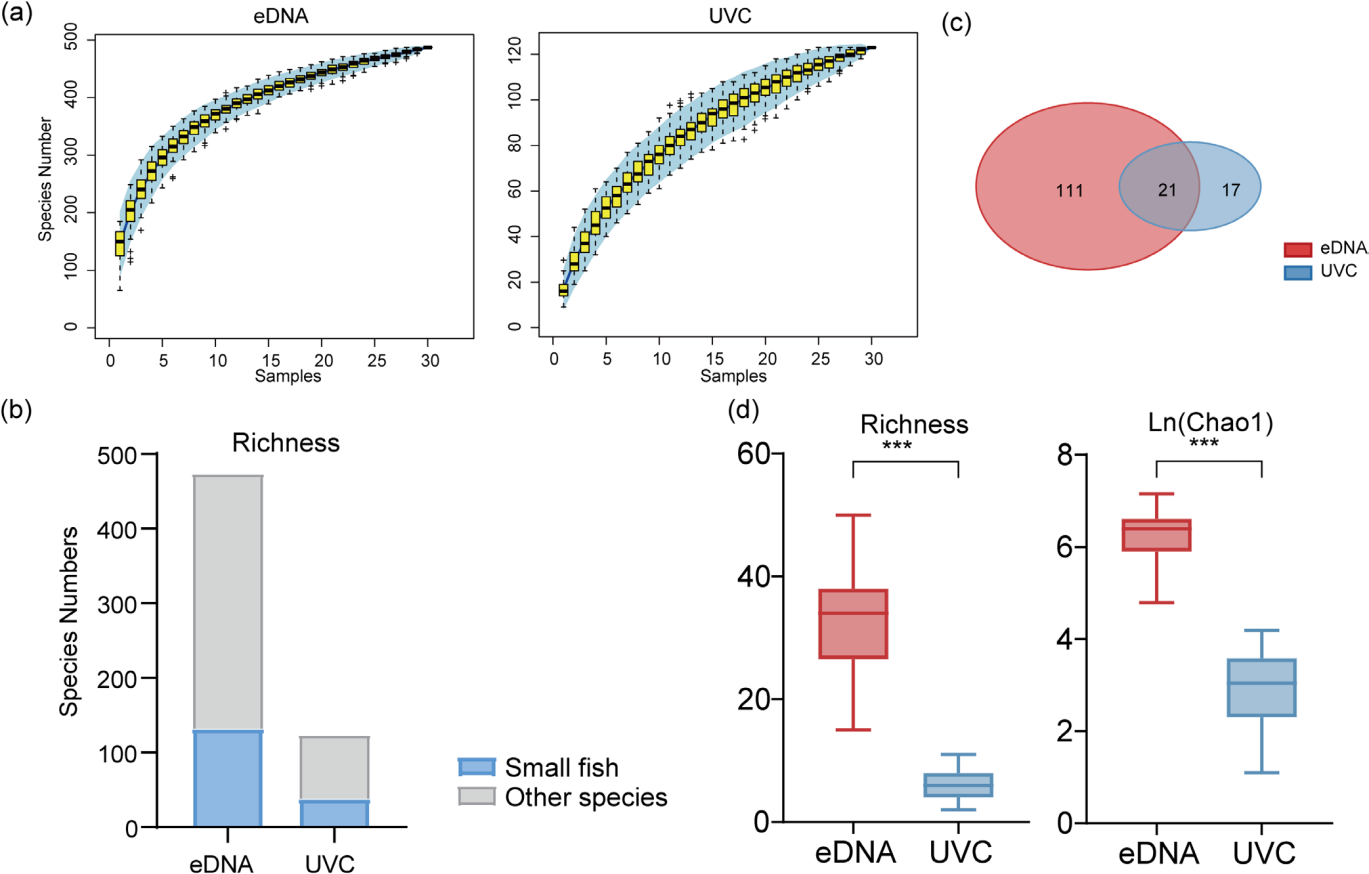
Comparative analysis of the efficacy of two sampling methodologies. (a) Species accumulation curves for eDNA samples and UVC surveys. (b) Comparative analysis of small fish proportions in eDNA and UVC sampling results. (c) The degree of overlap in small fish species detected between eDNA and UVC. (d) Comparative analysis of small fish species richness and Chao1 index detected by eDNA and UVC methodologies (***p < 0.001).

A total of 5,823,431 high‐quality 12S rRNA gene sequences were obtained from 29 eDNA samples (range: 108,595–247,185 per sample). The eDNA method detected 473 fish species, including 132 small fish species across 10 sampling sites at Meiji reef (Table S4); the UVC method recorded 123 species, of which 38 were small fish species (Table S5). Species‐rarefied curves of eDNA metabarcoding approached a plateau, indicating that the current sequencing depth was adequate to capture the fish species diversity in the samples. In contrast, the species‐rarefied curves for UVC data of identified individuals remained non‐saturating (Figures S2 and S3). Separately, the species accumulation curves of samples for both eDNA and UVC nearly reached a plateau (Figure 2a). The proportions of small fish detected by the two methods were 27.9% and 30.9%, respectively (Figure 2b). There were 21 species of small fish that were detected by eDNA and also identified through UVC records. These species include *Chrysiptera brownriggii* . In contrast, UVC uniquely detected 17 species not captured by eDNA, like *Pomacentrus lepidogenys* (Figure 2c). eDNA metabarcoding detected 55.3% of the small fish species recorded by UVC (A complete list of species detections is available in Table S3). Overall, eDNA metabarcoding yielded significantly higher estimates of small fish species richness and Chao1 index than UVC (*p* < 0.001), demonstrating its superior sensitivity for detecting small fish species (Figure 2d).

### 3.3 Differences in Small Fish Communities Between Regions

**FIGURE 3:**
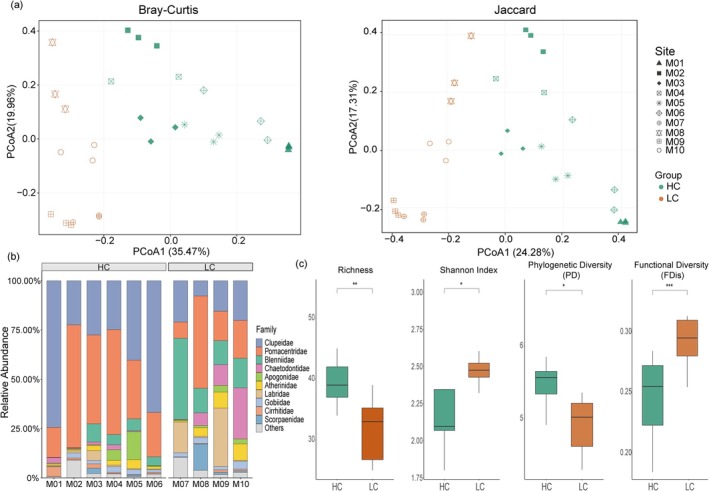
Comparative analysis of species composition among sampling site groupings based on the eDNA result. (a) β‐diversity analysis of small fish community across sampling sites using Bray‐Curtis and Jaccard dissimilarity matrices, where red denotes HC, blue represents LC, and distinct symbols indicate different sampling sites. (b) Comparative analysis of the top 10 most abundant families in terms of total abundance between HC and LC. (c) Comparative analysis of species richness, Shannon index, functional dispersion, and phylogenetic diversity index between HC and LC groups (***p < 0.001; **p < 0.01; *p < 0.05).

We examined differences in small fish communities between the HC and LC regions. Principal coordinates analysis (PCoA) revealed a significant distinction in fish species composition between the HC and LC groups (PERMANOVA, *p* < 0.001; Figure 3a). Further analysis of the top 10 most abundant families indicated distinct differences in relative proportions in the LC group (Figure 3b). Specifically, the HC areas were dominated by Pomacentridae and Clupeidae. In contrast, the LC areas showed a marked increase in the proportion of Blenniidae and Labridae, indicating a shift in community composition in response to reduced coral cover. In UVC observations, Pomacentridae was the dominant group, whereas Clupeidae was not recorded (Figure S4). However, Clupeidae was documented in separate imagery obtained after the UVC survey (Figure S9). In terms of alpha diversity, the HC group showed significantly higher phylogenetic diversity (PD) and species richness than the LC group. However, the Shannon index and functional dispersion (FDis) were substantially lower in the HC group (Figure 3c). The UVC‐based diversity analysis revealed that Pomacentridae was the dominant family in absolute terms. The Richness, Chao1 index, and Shannon index of HC were significantly higher than those of LC (*p* < 0.05), with a notable difference in community β‐diversity (*p* < 0.01) (Figures S5–S7).

### 3.4 Assembly Mechanisms of Small Fish Communities Along Coral Cover Gradients

**FIGURE 4:**
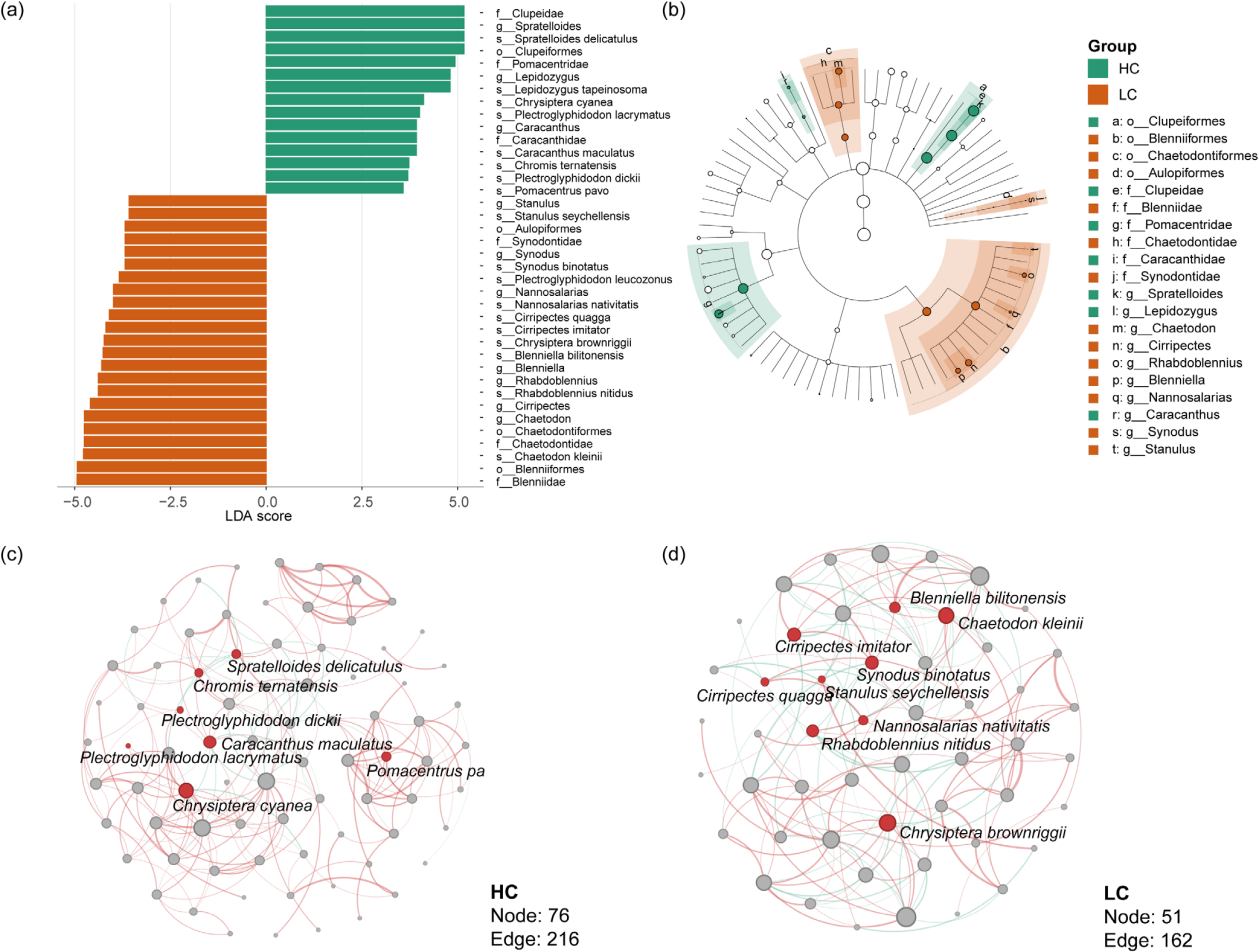
Indicator species and co‐occurrence networks of small fish communities from HC and LC. (a, b) Phylogenetic distribution of indicator species (identified by LEfSe) between HC and LC. Panel (a) shows the LDA scores of indicator species enriched in each group, and panel (b) displays their phylogenetic relationships. Co‐occurrence networks of small fish communities in the HC (c) and LC (d) groups based on Spearman correlation. Nodes represent species, with differential species highlighted in red. Edges represent significant correlations (red: Positive; green: Negative; *p* < 0.05), with thickness proportional to the strength of the correlation.

Furthermore, we investigated the underlying factors contributing to differences in small fish communities between HC and LC. LEfSe results reveal indicator species that are differentially enriched in HC and LC environments, respectively (Figure 4a,b). The LC environment exhibits an enrichment of 10 indicator species: *Chaetodon kleinii* , *Plectroglyphidodon leucozonus* , *C. brownriggii* , *Synodus binotatus* , *Blenniella bilitonensis* , *Cirripectes imitator* , *Cirripectes quagga* , *Nannosalarias nativitatis* , *Rhabdoblennius nitidus* , and *Stanulus seychellensis* . In contrast, the HC environment is associated with an enrichment of eight species: *Chromis ternatensis* , *Lepidozygus tapeinosoma* , *Plectroglyphidodon dickii*, *Plectroglyphidodon lacrymatus*, *Chrysiptera cyanea*, *Pomacentrus pavo* , *Caracanthus maculatus* , and *Spratelloides delicatulus*. Ecological networks of small fish communities indicate that indicator species occupied key nodes within the network (Figure 4c,d). Compared with HC, the LC network exhibited fewer nodes and a tendency toward structural simplification. The modularity index exceeding 0.4 indicated that both communities displayed distinct modular structures. Both the modularity index (HC: 0.605, LC: 0.505) and the module coefficient (HC: 0.509, LC: 0.477) of the HC group are higher than those of the LC group, indicating that species in this environment tend to form tightly interconnected, highly specialized modules. The robustness of HC (0.835) is slightly lower than that of LC (0.848), though the overall difference is not statistically significant.

**FIGURE 5:**
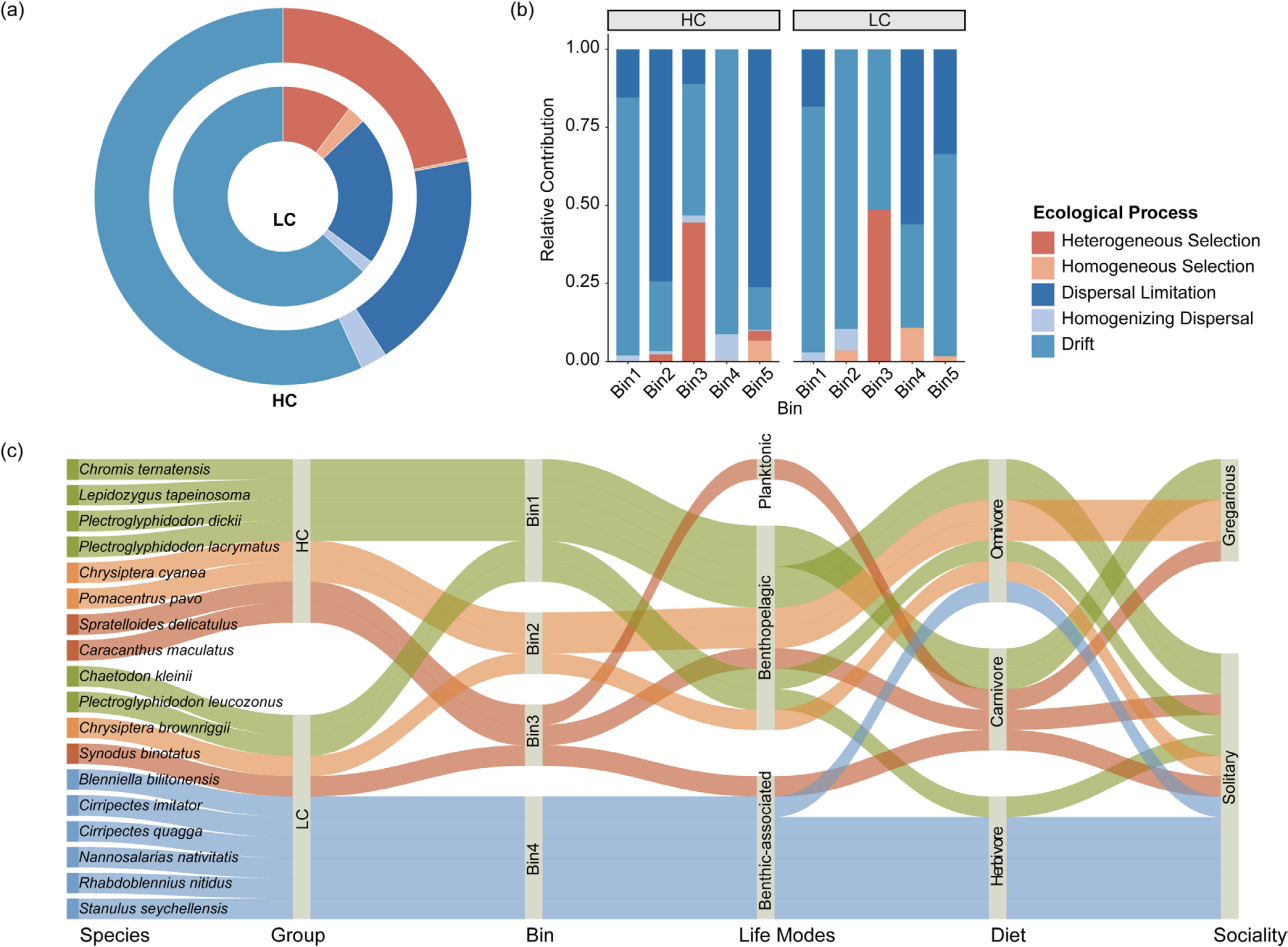
iCAMP results elucidate the distinct mechanisms underlying the assembly of small fish communities between HC and LC. (a) The ecological process contributes to the formation of small fish communities in HC and LC. (b) Dynamics of the dominant ecological processes within each phylogenetic bin of HC and LC. (c) Distribution of indicator species across the phylogenetic bins and their associated ecological traits.

The iCAMP analysis dissects the underlying ecological processes. Our results indicate that in the LC area, ecological drift (DR) was the dominant assembly process (63.1%), whereas the overall contribution of deterministic processes was substantially lower (approximately 13%; Figure 5a). In the HC area, the effect of ecological drift decreased, and heterogeneous selection (HeS) became the second most crucial process. Notably, dispersal limitation (DL) was a key assembly process influencing community structure in both areas. iCAMP partitioned the entire community into five ecological bins (Figure 5b). The indicator species identified by LEfSe analysis were assigned to Bins 1–4 (Figure 5c). Based on the ecological process analysis, distinct assembly patterns were identified across the different bins. In Bin1 and Bin4, DR remained the dominant process in both LC and HC habitats (78.7%–82.6%), with indicator species such as *C. kleinii* and *P. leucozonus* are characterized as generalists exhibiting low coral dependency. In Bin2 and Bin5, the dominant process shifted from DR (89.6% and 64.7%, respectively) in LC to DL (74.3% and 76.2%, respectively) in HC. This shift was accompanied by increased contributions from both heterogeneous (HeS) and homogeneous (HoS) selection. These bins included species such as *C. cyanea* and *P. pavo*, whose distributions are constrained by coral structural complexity and by their intrinsic dispersal capacity. Bin3 was predominantly shaped by environmental selection, with HeS (48.6%) and DL (51.4%) governing its structure in low coral cover, while HeS (44.5%) remained influential under high coral cover. Indicator species in this bin, including *S. binotatus* and *C. maculatus*, displayed heightened sensitivity to environmental filters. Overall, the ecological traits (e.g., life mode, diet, sociality) of the indicator species aligned with the inferred assembly mechanisms of their respective modules.

### 3.5 Eco‐Environmental Factors Influence the Distribution of Small Fish

**FIGURE 6:**
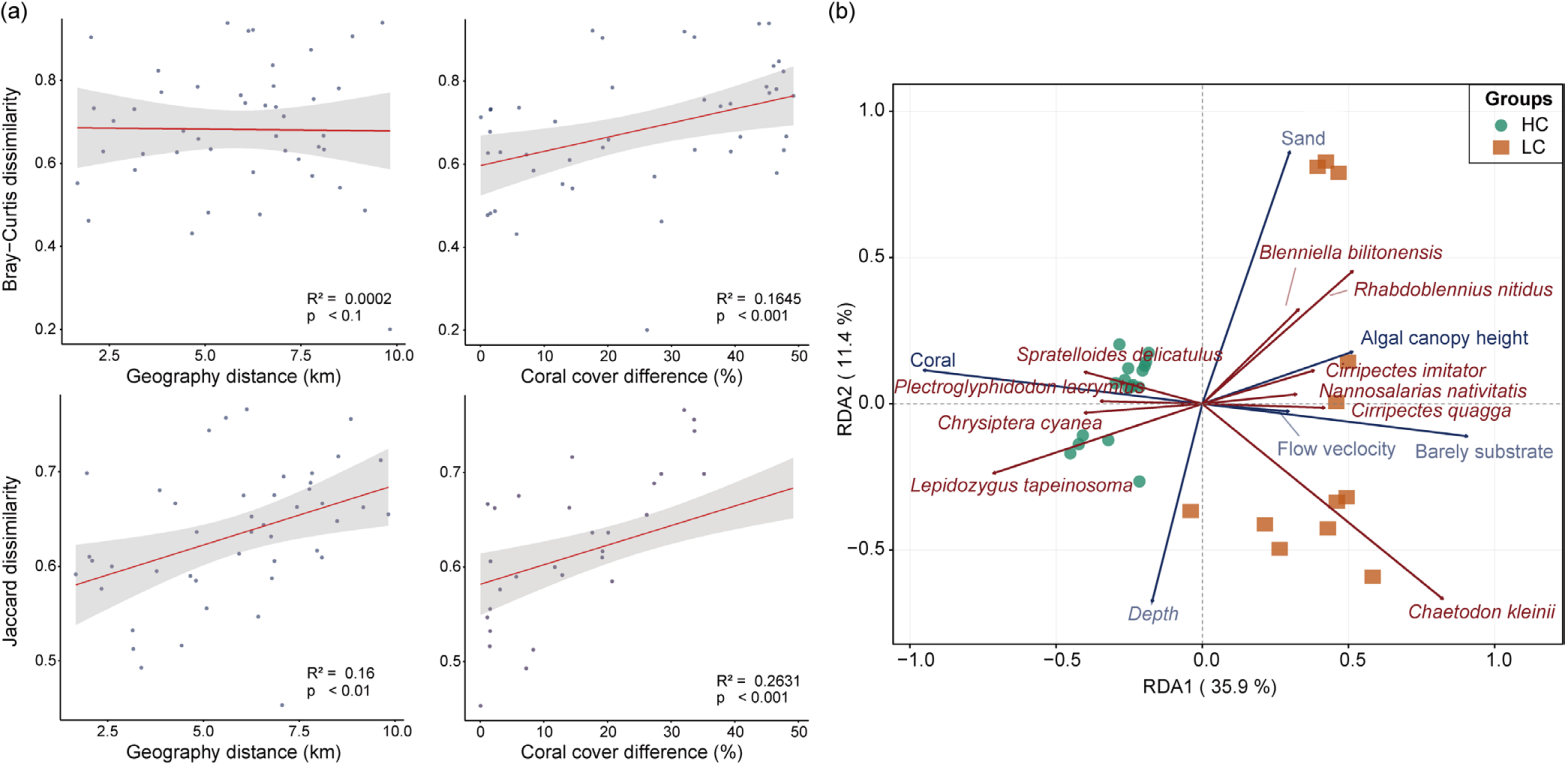
Impact of ecological factors on the distribution of small fish communities (a) Linear analysis of small fish community dissimilarity based on eDNA in relation to geographic distance and coral coverage differences. (b) RDA analysis showed that distinct ecological factors drive the communities from both HC and LC habitats, and the indicator species shaping their community structures also differ. Different colors represent HC and LC, respectively.

Linear regression analysis of small fish community diversity based on eDNA, in relation to geographical distance and coral cover, demonstrates that dissimilarity in small fish communities increases with rising differences in coral cover (Figure 6a), a phenomenon further corroborated by UVC results (Figure S8). Redundancy analysis was conducted on indicator species and remaining eco‐environmental factors. The first two axes of RDA jointly explained 47.3% of the species‐environment relationship. The HC and LC samples showed a distribution trend along the RDA1 axis in the ordination diagram, from high coral cover habitats to sand or barely substrates. There were 10 indicator species with absolute values of the species‐environment correlations in the RDA exceeding 0.2 (Figure 6b). At the positive end of RDA1, benthic trophic groups adapted to reef‐sand habitats clustered, including scraper species that feed on algae and attached organisms on reef surfaces ( *N. nativitatis* , *R. nitidus*, *C. quagga*, *C. imitator* ), as well as generalist species with flexible diets that can widely utilize various benthic resources (*C*. *kleinii*). Additionally, *B. bilitonensis* showed a specific preference for the mixed rubble‐sand habitat and has a relatively high correlation with sandy substrates. At the negative end of RDA1, species associated with structurally complex coral habitats were distributed, including *S. delicatulus*, as well as coral‐dependent species such as *L. tapeinosoma* , *C. cyanea*, and *P. lacrymatus*, which rely on coral structure for foraging and shelter.

## 4 Discussion

Our research represents the first integration of eDNA technology with UVC to detect small fish communities within the Meiji reef atoll of the Nansha Islands. This systematic investigation elucidates the biodiversity and distribution patterns of small fish communities across gradients of coral cover. Our findings substantiate the high sensitivity and effectiveness of eDNA for detecting small fish species, supporting its application in studies of small fish communities. Furthermore, we found that the role of deterministic processes in small fish community assembly increases with changes in coral cover, highlighting the ecological importance of coral habitat structure in shaping these communities.

Our data reveal a key methodological difference: while the eDNA rarefaction curve reached saturation (Figure S3), confirming adequate sequencing depth, the UVC curve did not plateau (Figure S2), suggesting that additional visual surveys could yield additional species. Crucially, however, species accumulation curves for the entire reef study demonstrated that both methods nearly achieved an adequate sample coverage (Figure 2a). eDNA showed higher detection sensitivity for some cryptic species, such as bottom‐dwelling Blenniidae with color highly integrated with the background, which are easily missed in visual monitoring (Table S3), whereas UVC is constrained by observer bias, fish behavior, and habitat complexity, which may result in the underestimation of small fish diversity. In addition, eDNA technology enables the detection of small fish inhabiting the upper water layers of coral reef areas, such as *S. delicatulus*, which often occurs in large aggregations near the water surface. Due to the resolution limitations of UVC equipment, this species was not accurately recorded during field surveys. The reliability of eDNA for detecting this species was confirmed through additional field surveys and specimen collection (Figure S9, Table S3). While eDNA metabarcoding consistently outperforms UVC in species detection, the two methods are highly complementary, capturing distinct datasets of community structure (Muenzel et al. 2024). However, eDNA failed to detect 20 small fish species that UVC recorded. This limitation common to eDNA metabarcoding can be attributed to several factors, including eDNA degradation, dilution effects, low species biomass, and insufficient coverage in reference databases (Curtis et al. 2021; Strickler et al. 2015; Zhang et al. 2022). Additionally, potential contamination during sampling and laboratory processing may further compromise detection accuracy. Therefore, stringent contamination control, optimized bioinformatics protocols, and continued expansion of reference databases are critical to improving the reliability and comprehensiveness of eDNA‐based small fish biodiversity monitoring.

Using eDNA metabarcoding, we detected significant differences in the small fish communities between the HC and LC habitats (Figure 3a). These findings indicate that eDNA can effectively capture spatial structural variations in small fish communities at the local scale, demonstrating its spatial fidelity as a reliable biomonitoring tool. At the family level, the HC community was predominantly composed of Pomacentridae and Clupeidae, with a higher relative read abundance. In contrast, the top 10 families in the LC community showed a more even distribution of reads, with notably increased proportions of Blenniidae and Labridae (Figure 3b). In the small fish communities of both coral reef habitats, reductions in coral cover led to significant decreases in species richness and phylogenetic diversity. Compared to the LC region, the HC region exhibited relatively higher species richness but a lower Shannon index, suggesting that one or several dominant species led to an uneven distribution of individuals among species (Figure 3c). Notably, despite declines in species and phylogenetic diversity, the community's functional diversity did not decrease synchronously, indicating that remaining species in degraded ecosystems can still maintain core ecological functions, consistent with previous research (Deksne et al. 2025; Xu et al. 2025). This phenomenon supports the idea that the functions of disappeared species are compensated by ecologically similar remaining species, allowing a few species to have a disproportionate impact on ecosystem functions and thereby helping maintain operational processes during environmental disturbances (Butler 2025). However, the continued reduction of functionally redundant species will weaken the ecosystem's buffering capacity, affecting its long‐term stability (Mouillot et al. 2014). Moreover, a key caveat of eDNA metabarcoding is that it primarily captures variations in relative sequence abundance, which may not directly translate to changes in absolute population sizes. For instance, increases in the relative abundance of specific taxa could result from declines in other species—even if the absolute abundance of the focal taxa remains unchanged—and vice versa. Thus, these patterns reflect relative shifts in species composition rather than absolute changes in fish biomass or individual density. The divergence in diversity metrics between eDNA and UVC stems from their distinct underlying data: UVC is based on individual counts, while eDNA read abundance correlates more strongly with organismal biomass. Consequently, the higher diversity indices from eDNA in the LC area may reflect the detection of low‐biomass groups rather than conclusively indicating higher fish diversity than in the HC area.

We found that coral cover drives small fish community composition primarily by serving as the dominant factor in habitat differentiation. The results demonstrate a pronounced south‐to‐north environmental gradient at Meiji reef, with the benthic habitat transitioning from coral‐dominated to barely substrates (Figure S1). In healthy coral reef ecosystems, the complex three‐dimensional structure, resource heterogeneity, and microhabitats generate strong environmental filtering that structures community composition by selecting for small fish adapted to specific habitats. In contrast, as reefs degrade, the breakdown of coral skeletons leads to habitat homogenization and resource uniformity, reducing the explanatory power of eco‐environmental factors in species distributions. In HC habitats, small fish species form tightly interconnected, highly specialized modules, which likely reflects finer niche differentiation within these complex habitats. In contrast, the more homogeneous environmental conditions in LC lead to a more uniform and loosely connected network structure, consistent with stochastic assembly processes (Figure 4c,d). Analysis of community assembly mechanisms revealed a shift in small fish communities from co‐dominance by environmental filtering and ecological drift to dominance by ecological drift. This transition reflects a weakening of the ecosystem's filtering function and a decrease in community predictability, indicating that stochastic processes increasingly determine species persistence under the pressure of coral reef degradation (Figure 5a,b). A similar transition in community assembly mechanisms has been documented in other coral systems, such as those in Mexico and Okinawa's Naha Bay (Jarquín‐Martínez et al. 2025; Kawade et al. 2025), implying that the shift from environmental filtering to stochastic dominance may be a universal phenomenon during coral degradation.

Our analysis of indicator species revealed that HC were characterized by species highly dependent on live coral structures or inhabiting the water column above healthy reefs (Figure 5c). For instance, *P. lacrymatus* and *P. dickii* complete their foraging and habitation within coral branches (Emslie et al. 2012; Tebbett et al. 2020). The schooling fish *C. ternatensis* relies on complex coral structures to evade predators (Wismer et al. 2019). Furthermore, the greater abundance of *S. delicatulus* in HC zones may be attributed to more robust food webs and greater availability of planktonic resources (Kingsford et al. 2022). In contrast, species composition in LC areas exhibits markedly different adaptive characteristics, with these groups generally showing lower dependence on live corals and greater adaptability to more open, structurally simpler habitats. Representative species include benthic Blennies inhabiting rock surfaces and capable of tolerating strong currents in rubble environments (Bennett‐Smith et al. 2025), as well as *P. leucozonus*, which exhibits broader dietary preferences and can survive under resource‐limited conditions (Alsaaq 2024). Additionally, the ambush predator *S. binotatus* utilizes the expansive sandy or rubble substrates of LC regions for hunting. In the absence of physical shelter provided by corals, cryptic coloration of fish in LC regions that closely matches the substrate provides crucial camouflage against predation pressure. Our study further reveals that, against the backdrop of increased overall community stochasticity, the small fish community responds more stochastically to environmental stress (Xu et al. 2025). This disparity may stem from their typical r‐selected life history strategies, which render them more sensitive to random birth‐death events. At the same time, their limited mobility makes their dispersal and settlement processes more susceptible to physical fluctuations such as water currents and substrate structures. Consequently, due to their rapid response to environmental fluctuations, small fish demonstrate unique advantages in reflecting local habitat conditions, positioning them as potential indicator taxa for monitoring regional ecological change.

A comprehensive elucidation of the distribution patterns of small fish communities is crucial for understanding the integrity and resilience of coral reef ecosystems (Chase et al. 2018, 2020; Shantz et al. 2023). However, current research on small fish communities primarily focuses on shallow waters, while their distribution and abundance in deeper zones remain poorly understood (Kane et al. 2014). Future studies should prioritize systematic investigations of small fish in the deep‐water zone to better reflect fish diversity and function in coral reef ecosystems. eDNA metabarcoding provides a robust foundation for such research. Its low cost and risk nature make it particularly well‐suited for monitoring small fish in deep‐water environments. With enhanced reference databases and optimized sampling protocols, eDNA technology is expected to become a pivotal tool for uncovering the dynamics and ecological functions of small fish communities. While the ecological functions of small fish on coral reefs remain incompletely understood, particular species, such as those in the Gobiidae family, have emerged as bioindicators of ecosystem health (Goatley et al. 2016). Moving forward, it is imperative to elucidate the mechanistic links between their functional traits, roles in community assembly, and critical ecosystem processes. Such a mechanistic understanding will provide a foundational basis for novel conservation strategies and the adequate long‐term protection of coral reef biodiversity.

## 5 Conclusion

The study identified 149 small fish species on the coral reefs of Meiji reef in the Nansha Islands, of which 132 were detected by eDNA metabarcoding. Compared with the UVC method, eDNA metabarcoding detected a greater number of small fish species, including rare and cryptic taxa that are difficult to observe using visual surveys. Coral cover significantly influenced the composition of small fish communities. The community network structure exhibited higher specificity and modularity in stable, healthy environments, relying heavily on keystone taxa. In contrast, it transitioned to a looser, more random system composed of opportunistic species in resource‐depauperate environments. Consequently, the indicator species supporting the ecological network shifted from coral‐dependent Pomacentridae and Clupeidae to Blenniidae, which rely less on live coral, reflecting the ecosystem simplification driven by the loss of structured coral habitat. As coral cover decreased, the community assembly shifted from being governed by a combination of deterministic filtering (environmental pressure) and stochastic effects to being dominated by stochastic processes. These findings collectively illustrate how declining coral cover weakens environmental filtering and strengthens stochastic dispersal, ultimately driving small fish communities toward a simpler, potentially unstable state.

## Author Contributions

**Daxiao Ling:** conceptualization (equal), data curation (supporting), formal analysis (lead), writing – original draft (lead). **Xianzhi Lin:** conceptualization (equal), data curation (lead), formal analysis (supporting), investigation (lead), validation (lead), writing – review and editing (supporting). **Yanyan Zhou:** data curation (supporting), investigation (supporting). **Yang Liu:** data curation (supporting), investigation (supporting). **Haoye Lin:** investigation (supporting). **Li Zhang:** conceptualization (equal), funding acquisition (lead), resources (lead), supervision (lead), writing – review and editing (equal).

## Funding

This work was supported by National Key Research and Development Program of China (2022‐20), Science and Technology Planning Project of Guangdong Province, China (2023B1212060047), the program of State Key Laboratory of Tropical Oceanography, South China Sea Institute of Oceanology, Chinese Academy of Sciences (SKLTO2025PT002), Hainan Provincial Natural Science Foundation of China (423CXTD392), and Special Fund of South China Sea Institute of Oceanology of the Chinese Academy of Sciences (SCSIO2023QY04).

## Ethics Statement

The fish study was reviewed and approved by the Laboratory Animal Management and Ethics Committee, South China Sea Institute of Oceanology, Chinese Academy of Sciences.

## Conflicts of Interest

The authors declare no conflicts of interest.

## Supporting information


**Figures S1–S9:** ece373553‐sup‐0001‐FigureS1‐S10‐TableS1‐S2.docx.
**Tables S1–S2:** ece373553‐sup‐0001‐FigureS1‐S10‐TableS1‐S2.docx.

**Table S3:** ece373553‐sup‐0002‐Table S5.xlsx.

**Table S4:** ece373553‐sup‐0003‐Table S4.xlsx.

**Table S5:** ece373553‐sup‐0004‐Table S3.xlsx.

## Data Availability

The raw short‐read DNA sequencing data were deposited in the NCBI Sequence Read Archive under the accession numbers SRR36105486–SRR36105455.
