# Supplementary material for: Shifts in Small Fish Community Caused by Coral Cover Variation at a Remote Reef in the South China Sea
Source: Ecol Evol. 2026 Apr 23;16(4):e73553. doi: 10.1002/ece3.73553 (PMC13105832; doi:10.1002/ece3.73553)
Supplement: Supplementary file 1 — Figures S1–S9: ece373553‐sup‐0001‐FigureS1‐S10‐TableS1‐S2.docx. Tables S1–S2: ece373553‐sup‐0001‐FigureS1‐S10‐TableS1‐S2.docx. [file ECE3-16-e73553-s003.docx]

**Supplementary material**

**Ecological factors**

The ecological factor data of the sampling sites are summarized in Table S1. Coral cover, Hard coral, Soft Coral, Bleaching Coral, Macroalgae, Sessile Organisms, Barely Substrate, Sand, and Algal turfs are cover rates (%). Coral cover denotes the total coral cover, which is the sum of the cover rates of hard corals, soft corals, and bleached corals. Hard corals encompass cover rates of Scleractinia, Tubipora musica, and Heliopora coerulea. Macroalgae are large algae with heights greater than 5 cm. Sessile organisms encompass cover rates of Milleporina, Actiniaria, and Porifera. Barely Substrate is defined as a hard substratum with a turf algae cover of less than 2 mm in height. Algal turfs refers to a mat-like community formed by diminutive algae less than 2 cm tall. Depth refers to the maximum depth of the water body in the sampling point area. The calculation method for the cover rate of a specific type of coral is (the total covered length of this type of coral under the quadrat / the total length of the quadrat) × 100%.

Principal Component Analysis (PCA) results indicate that coral cover constitutes the most significant ecological factor influencing the habitat characteristics of sampling sites. Based on coral cover, the sampling sites were categorized into two distinct groups: HC (High Coral Cover) and LC (Low Coral Cover). (Fig.S1). Correlation analysis of the measured ecological factors revealed statistically significant relationships between multiple pairs of variables, as shown in the heatmap (Fig. S7). The significant negative correlations between coral cover and barely Substrate cover (p < 0.001) and algal turfs cover indicate a displacement of these substrates by live corals. Coral cover was negatively correlated with water flow velocity, suggesting that the complex structure of coral colonies impedes water movement. Barely subtrate cover was positively correlated with algal canopy height (p < 0.05), indicating that areas lacking coral develop characteristics of an algal turf-dominated reef rock habitat. Collectively, the relationships among these eco-environmental factors demonstrate that the Meiji Reef area is an ecosystem in which the biological and physical environments are tightly coupled.

Analysis of Community Composition and Diversity Based on UVC Results

We evaluated the detection efficacy of UVC and analyzed community species diversity and composition based on the detection results. The rarefaction curve for eDNA data approached an asymptote, whereas the UVC data curve remained non-saturating (Fig. S2, S3). In community composition analysis, Pomacentridae constitutes the dominant species in absolute terms, which may be attributed to their vibrant coloration being more easily detected by UVC. (Fig. S4). The relative abundance of Blenniidae in LC is higher compared to that in HC. In subsequent analyses, the overall trend in β-diversity across sites was comparable to that observed in the eDNA results, with significant differences between sites (Fig. S5). The alpha diversity indices of the HC small fish community are significantly higher than those of LC, as evidenced by the richness, Chao1 index, and Shannon index (Fig. S6). These findings provide robust substantiation for the eDNA detection results.


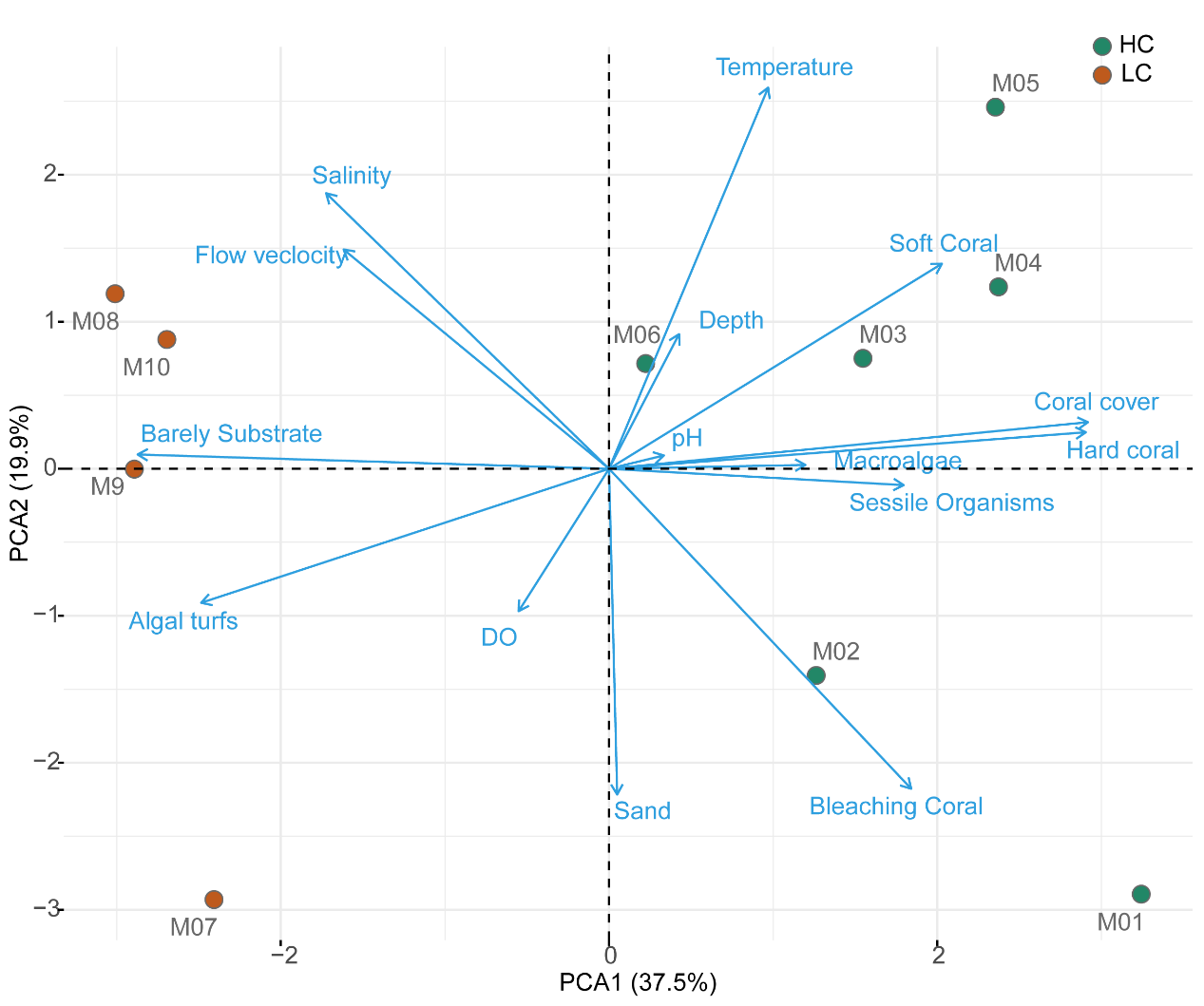


**Fig. S1** Principal Component Analysis (PCA) was conducted to examine the relationship between various ecological factors and the samples. The arrows represent distinct ecological factors, including temperature, depth, salinity, pH, DO, sand, flow velocity, barely substrate, algal turfs, sessile organisms, macroalgae, hard coral, soft coral, bleaching coral, and coral cover. The sample points, differentiated by shape and color, correspond to HC and LC, respectively.


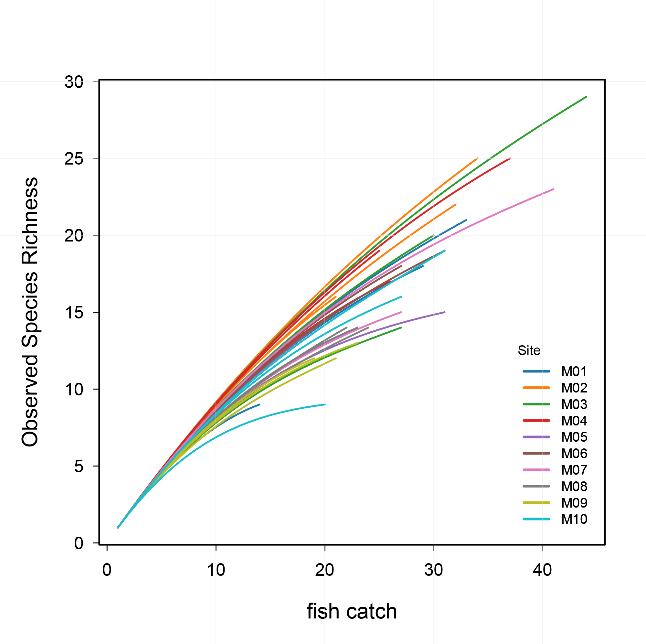


**Fig. S2**. Species dilution curve based on UVC Results. The x-axis represents the number of fish captured by UVC, while the y-axis indicates the number of species detected. Different colors denote distinct sampling stations.


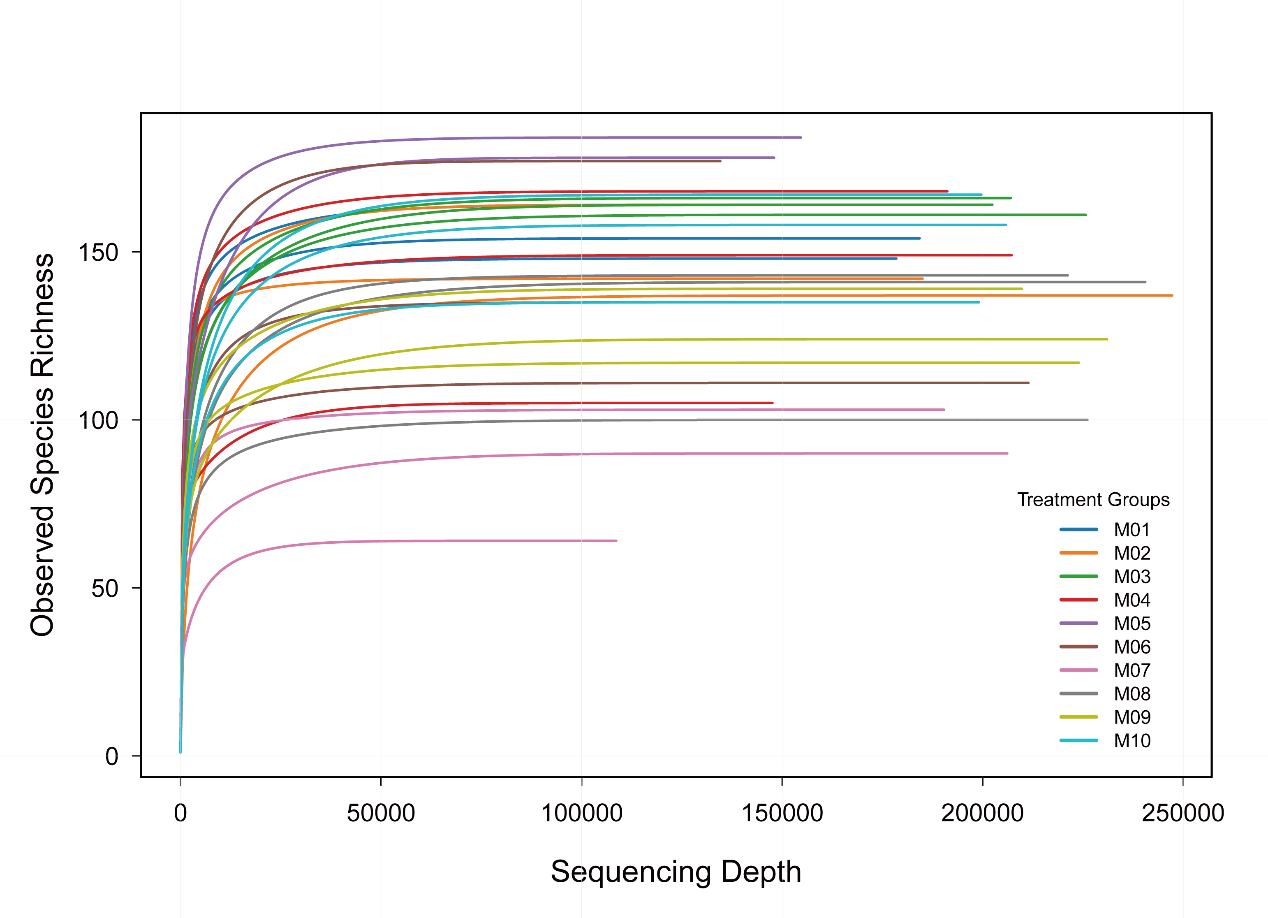


**Fig. S3.** Species dilution curve based on eDNA Results.


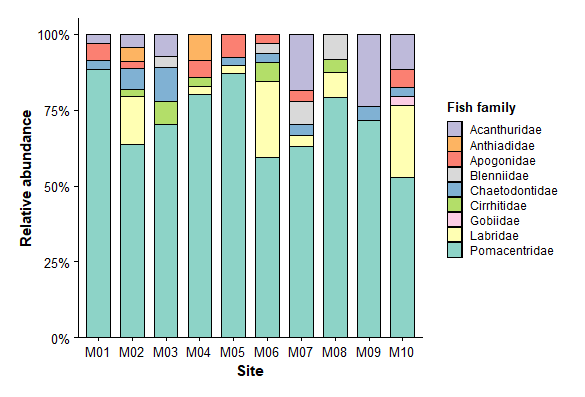


**Fig. S4.** The bar chart illustrates the relative abundance of small fish species at various stations across the scientific level, based on UVC survey results.


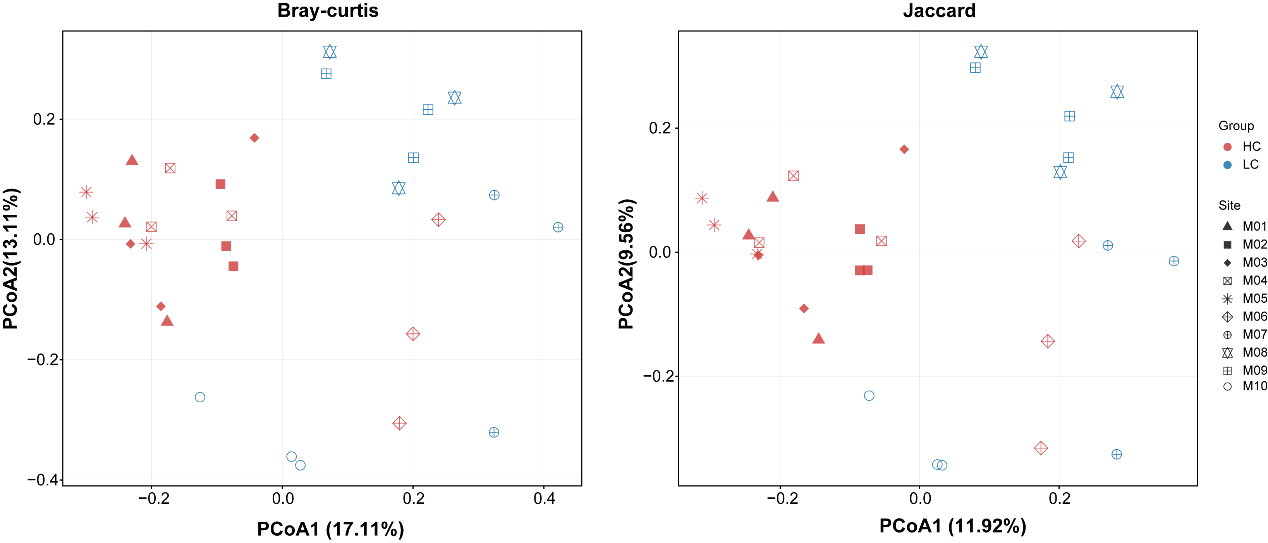


**Fig. S5.** β-diversity analysis of small fish communities across sampling sites based on UVC detection, where red denotes HC, blue represents LC, and distinct symbols indicate different sampling locations.


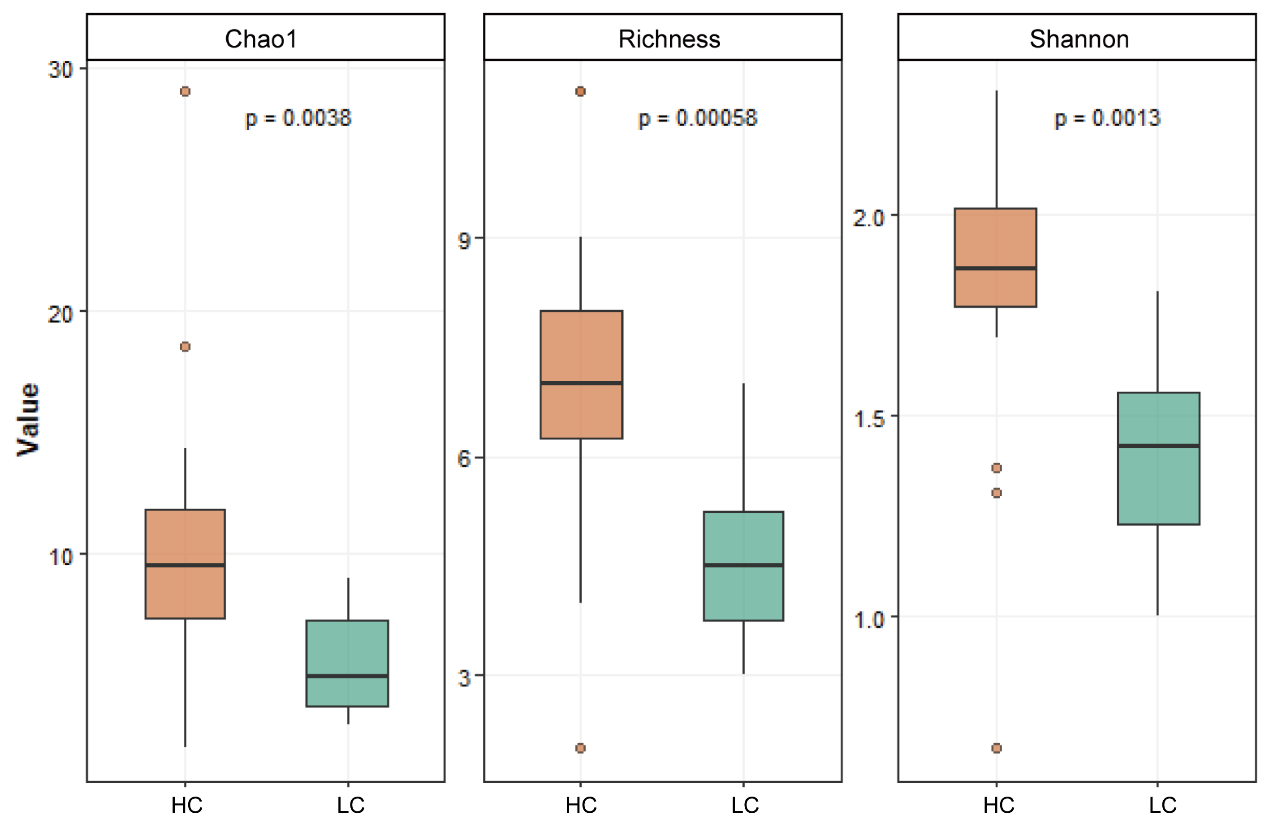


**Fig. S6.** Comparative analysis of Chao1 index, richness, and Shannon index between HC and LC groups based on UVC detection(***, P ＜ 0.001; **, P ＜ 0.01; *, P ＜ 0.05).


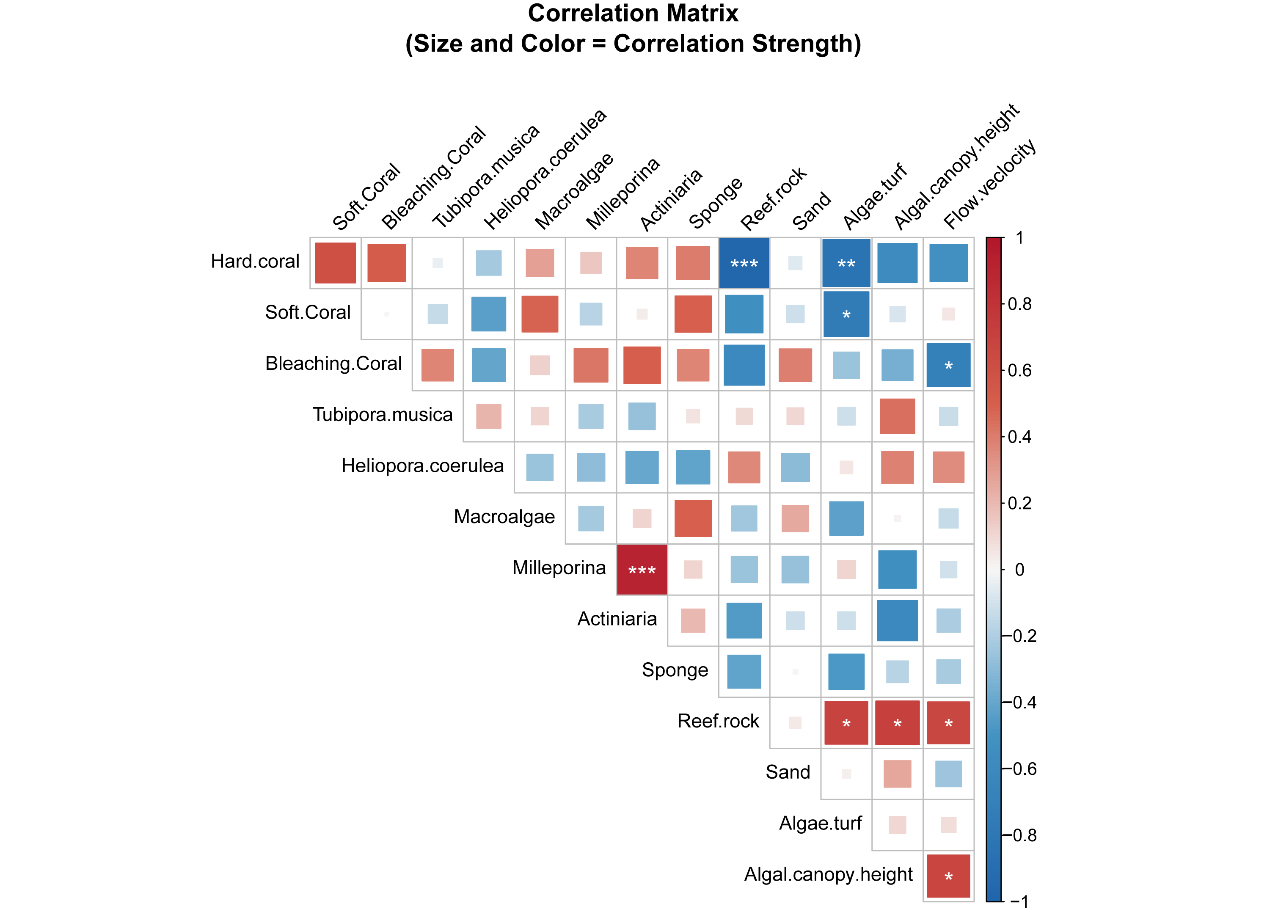


**Fig. S7.** Correlation Analysis Heatmap of Ecological Factors


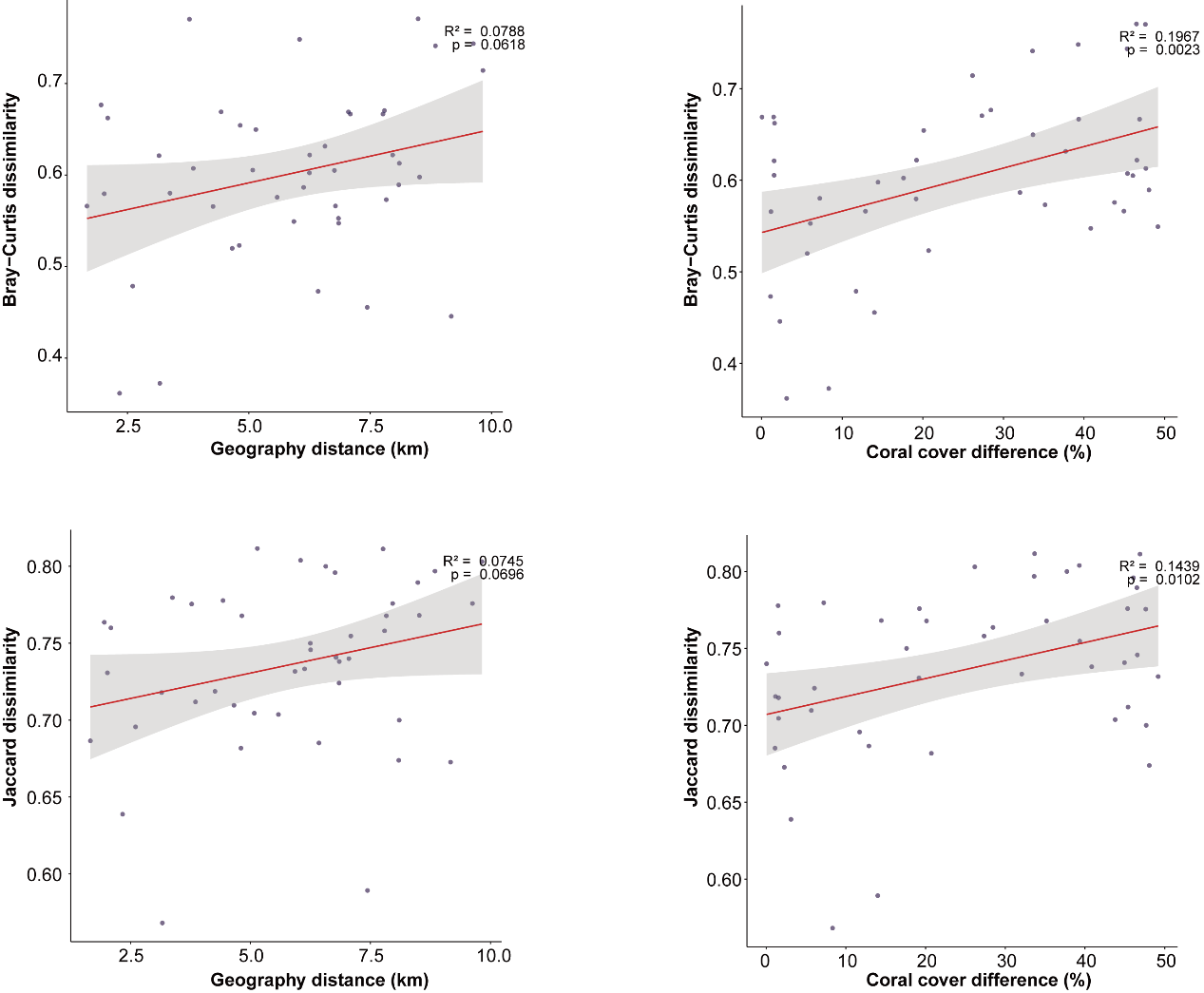


**Fig. S8.** Linear analysis of small fish community diversity based on UVC in relation to geographic distance and coral cover.


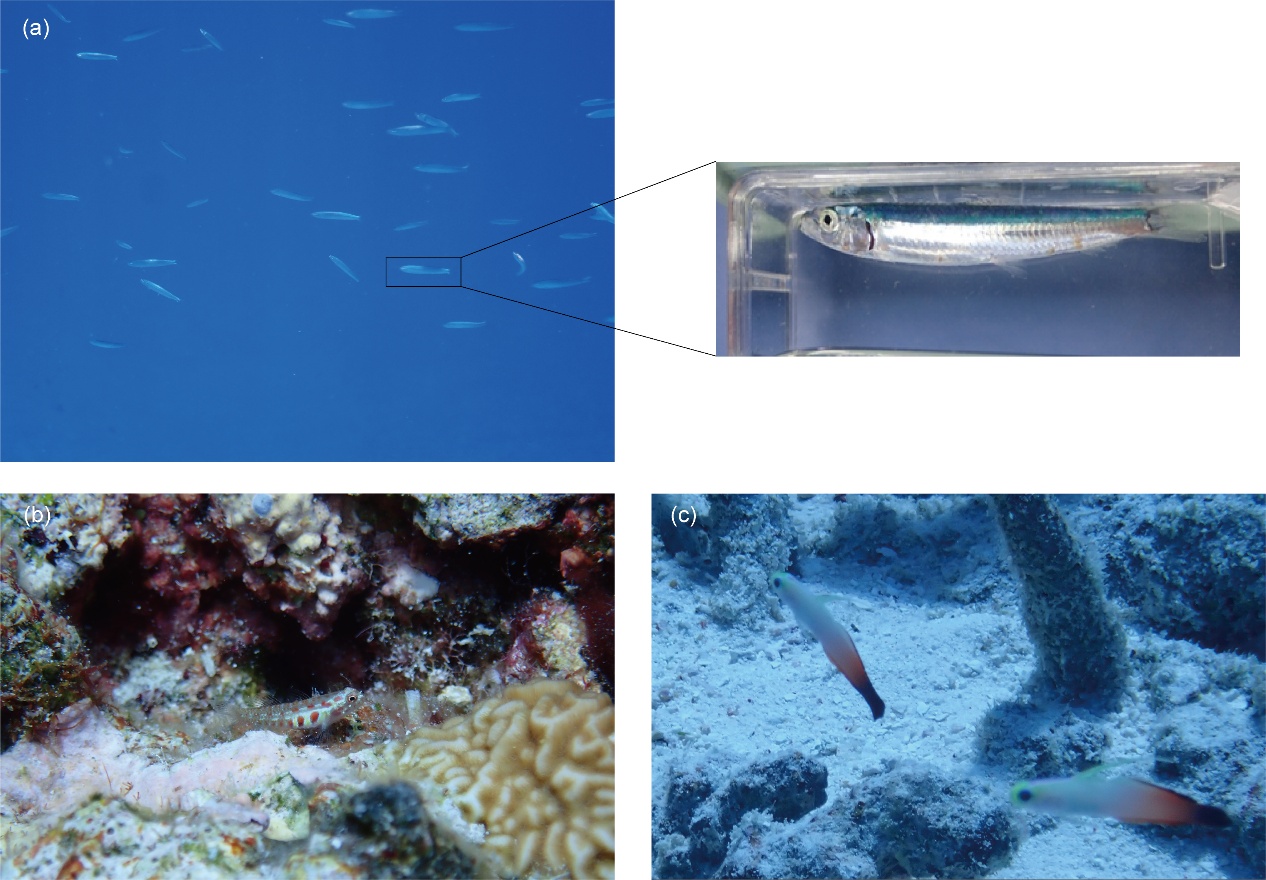


**Fig. S9.** The small fish species detected by eDNA were not found in the UVC. All the aforementioned photographs were captured individually after the UVC acquisition process. (a) *Spratelloides delicatulus* (b) *Eviota albolineata* (c) *Nemateleotris magnifica*

| **Sample** | **M01** | **M02** | **M03** | **M04** | **M05** | **M06** | **M07** | **M08** | **M09** | **M10** |
| --- | --- | --- | --- | --- | --- | --- | --- | --- | --- | --- |
| Depth (m) | 9.00 | 7.80 | 9.20 | 8.00 | 7.40 | 8.30 | 5.00 | 8.70 | 5.00 | 12.0 |
| Temperature (℃) | 29.2 | 29.5 | 30.6 | 30.0 | 30.6 | 29.9 | 29.0 | 29.8 | 29.6 | 29.8 |
| Salinity | 32.9 | 32.9 | 33.1 | 33.2 | 33.0 | 33.1 | 33.0 | 33.3 | 33.2 | 33.3 |
| Dissolved Oxygen (Mg/L) | 5.47 | 4.91 | 6.58 | 4.91 | 3.95 | 5.29 | 5.50 | 5.37 | 5.47 | 5.10 |
| pH | 8.06 | 8.50 | 8.16 | 8.11 | 8.13 | 8.11 | 8.01 | 8.14 | 8.15 | 8.17 |
| Coral cover (%) | 48.1 | 36.6 | 49.8 | 42.6 | 51.0 | 22.4 | 3.22 | 1.89 | 5.06 | 3.44 |
| Hard coral (%) | 46.9 | 36.4 | 49.4 | 39.9 | 47.8 | 20. | 3.17 | 1.78 | 4.94 | 3.44 |
| Soft Coral (%) | 1.17 | 0.11 | 0.39 | 2.67 | 3.17 | 1.61 | 0.06 | 0.11 | 0.11 | 0.00 |
| Bleaching Coral (%) | 0.83 | 0.61 | 0.28 | 0.22 | 0.17 | 0.28 | 0.33 | 0.11 | 0.06 | 0.06 |
| Macroalgae (%) | 0.17 | 0.00 | 0.00 | 0.50 | 0.00 | 0.00 | 0.06 | 0.06 | 0.00 | 0.00 |
| Sessile Organisms (%) | 2.44 | 1.33 | 0.94 | 2.89 | 0.39 | 3.72 | 0.11 | 0.17 | 0.00 | 0.39 |
| Barely Substrate (%) | 38.6 | 44.8 | 43.5 | 48.9 | 42.8 | 61.4 | 74.6 | 83.1 | 72.4 | 69.1 |
| Sand (%) | 0.78 | 0.00 | 0.22 | 0.39 | 0.00 | 0.00 | 1.50 | 0.00 | 0.00 | 0.00 |
| Algal turfs (%) | 8.72 | 16.7 | 5.22 | 4.39 | 3.39 | 11.8 | 20.1 | 14.6 | 22.5 | 26.9 |
| Flow velocity (mm/s) | 41.3 | 247 | 247 | 337 | 391 | 445 | 381 | 547 | 407 | 229 |

Table S1 | Ecological factors of sampling sites

| **Abbreviations** | **Full title** |
| --- | --- |
| DNA | deoxyribonucleic acid |
| rRNA | ribosomal RNA |
| eDNA | Environmental DNA |
| UVC | Underwater Visual Census |
| SCUBA | Self-Contained Underwater Breathing Apparatus |
| DO | dissolved oxygen |
| MCE | mixed cellulose ester |
| CTAB | cetyltrimethylammonium bromide |
| PCA | principal component analysis |
| HC | High Coral Cover |
| LC | Low Coral Cover |
| PCR | polymerase chain reaction |
| ASV | amplicon sequence variants |
| maxEE | maximum expected error |
| TD | taxonomic alpha diversity |
| FD | functional alpha diversity |
| Fric | functional richness index |
| PD | phylogenetic alpha diversity |
| PCoA | Principal Coordinates Analysis |
| LDA | linear discriminant analysis |
| LEFSe | linear discriminant analysis and effect size analysis |
| iCAMP | Infer Community Assembly Mechanisms by Phylogenetic bin-based null model |
| HoS | homogenizing selection |
| HeS | heterogeneous selection |
| DL | dispersal limitation |
| HD | homogeneous dispersal |
| DR | ecological drift |
| RDA | redundancy analysis |

Table S2 | List of abbreviations
